# Supplementary material for: The causal relationship between the human gut microbiota and pyogenic arthritis: a Mendelian randomization study
Source: Front Cell Infect Microbiol. 2024 Nov 26;14:1452480. doi: 10.3389/fcimb.2024.1452480 (PMC11629706; doi:10.3389/fcimb.2024.1452480)
Supplement: Supplementary file 1 [file DataSheet1.zip › mendelian test/ebi-a-GCST90027655.csv_forest.pdf]

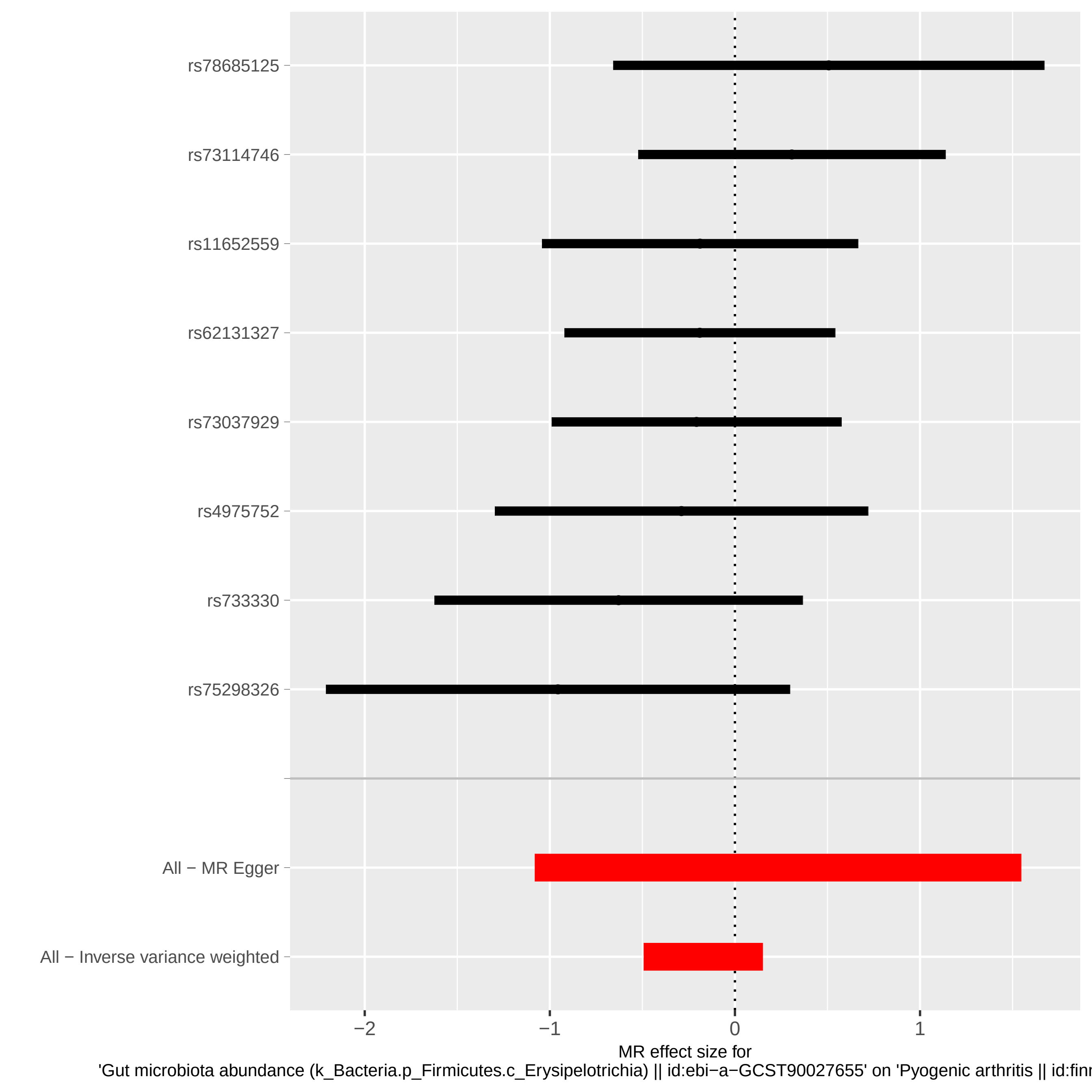

rs78685125
rs73114746
rs11652559
rs62131327
rs73037929
rs4975752
rs733330
rs75298326
All − MR Egger
All − Inverse variance weighted
−2
−1
0
1
MR effect size for
'Gut microbiota abundance (k_Bacteria.p_Firmicutes.c_Erysipelotrichia) || id:ebi−a−GCST90027655' on 'Pyogenic arthritis || id:finn
